# Effects of Decreased Occlusal Loading during Growth on the Mandibular Bone Characteristics

**DOI:** 10.1371/journal.pone.0129290

**Published:** 2015-06-10

**Authors:** Natsuko Hichijo, Eiji Tanaka, Nobuhiko Kawai, Leo J. van Ruijven, Geerling E. J. Langenbach

**Affiliations:** 1 Department of Orthodontics and Dentofacial Orthopedics, Tokushima University Graduate School of Oral Sciences, Tokushima, Japan; 2 Department of Orthodontics and Dentofacial Orthopedics, Institute of Biomedical Sciences, Tokushima University Graduate School, Tokushima, Japan; 3 Department of Orthodontics, Faculty of Dentistry, King Abdulaziz University, Jeddah, Saudi Arabia; 4 Department of Oral Cell Biology and Functional Anatomy, Academic Centre for Dentistry Amsterdam (ACTA), Research Institute MOVE, University of Amsterdam and VU University Amsterdam, Amsterdam, Netherlands; Georgia Regents University, College of Dental Medicine, UNITED STATES

## Abstract

**Background:**

Bone mass and mineralization are largely influenced by loading. The purpose of this study was to evaluate the reaction of the entire mandibular bone in response to decreased load during growth. It is hypothesized that decreased muscular loading will lead to bone changes as seen during disuse, i.e. loss of bone mass.

**Methods and Findings:**

Ten 21-day-old Wistar strain male rats were divided into two groups (each n=5) and fed on either a hard- or soft-diet for 11 weeks. Micro-computed tomography was used for the investigation of bone mineralization, bone volume, bone volume fraction (BV/TV) and morphological analysis. Mandibular mineralization patterns were very consistent, showing a lower degree of mineralization in the ramus than in the corpus. In the soft-diet group, mineralization below the molars was significantly increased (p<0.05) compared to the hard diet group. Also, bone volume and BV/TV of the condyle and the masseter attachment were decreased in the soft-diet group (p<0.05). Morphological analysis showed inhibited growth of the ramus in the soft-diet group (p<0.05).

**Conclusion:**

Decreased loading by a soft diet causes significant changes in the mandible. However, these changes are very region-specific, probably depending on the alterations in the local loading regime. The results suggest that muscle activity during growth is very important for bone quality and morphology.

## Introduction

Bone characteristics are largely influenced by the loads imposed upon them. Increased natural loads increase the bone mass [1,2]. Bone loss can be seen in individuals with decreased loading pattern [3,4]. In vivo mechanical loading of bone tissue (eg three-point bending) has shown that loading amplitude [5], cycle number [6] and frequency [7] are important factors in bone adaptation. Indirect evidence shows that the majority of the natural loadings are generated by muscles, and that alterations in these natural muscles forces are important for bone adaptation [8,9,10]. This adaptation can be local, as indicated by highly mineralized bones in the playing arm of tennis players [11] or systemic impact physiological loads [12]. Paralysis has been shown to lead to bone mass decrease [13,14]. In the craniofacial region, studies on the effect of changed loading patterns show comparable but specific reactions. Myotonic dystrophy patients, who have a lower masseter muscle activity, show some atypical mandibular forms, characterized by a large mandibular plane angle and changes of shape regarding the temporomandibular joint [15,16,17]. A soft diet during development causes a reduction in jaw bone development [18,19,20] and only a small change in the daily jaw muscle activity [18].

The purpose of this study was to investigate the regional reactions of the mandibular bone (including its form, and local bone volumes and mineralization degrees) in response to a, in time, limited decrease in daily load during growth. For this, the food consistency was decreased, assumingly resulting in a decreased masticatory muscle activity and occlusal load. We hypothesized that the decreased mandibular loading will result in a loss of bone mass, changes as seen during disuse.

## Materials and Methods

### Experimental animals

Ten Wistar strain male rats at the age of three-weeks were randomly divided into hard-diet and soft-diet groups (both n = 5). The use of a single gender was an attempt to eliminate any variation in bone characteristics due to sexual dimorphism. The hard-diet group was fed on an ordinary pellet (CE-2, CLEA Japan Inc., Tokyo, Japan), while a powder diet that contained the same constituents was used in the soft-diet group. Body weight was monitored once a week.

At 13-week-old, the animals were killed with an overdose of sodium pentobarbital (Nembutal; Dinabott, Osaka, Japan). The right mandibles were removed and examined by a micro-computed tomography system (micro-CT) for bone density, mineralization and morphometric analyses. These specimens were stored in 70% ethanol for maximally one month.

The protocol of the experiment was approved by the Animal Care and Use Committee at the Tokushima University.

### Bones

#### Mineralization, bone volume and bone volume fraction (BV/TV)

**Fig 1:**
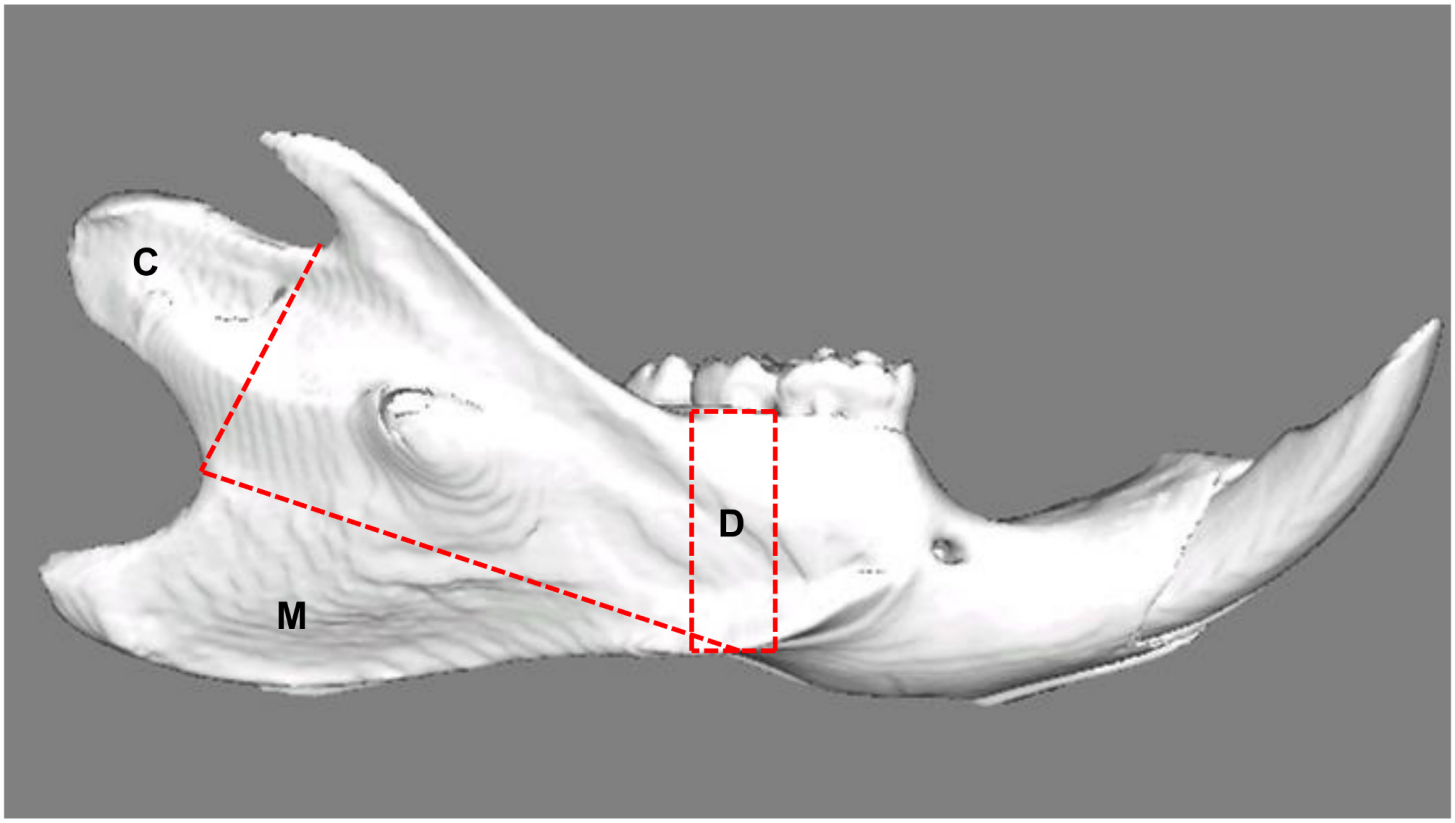
Lateral view of a reconstructed right hemimandible showing the volumes of interest. C: Condyle; Selection is defined by the line connecting the two notches superior and inferior of this area. M: Attachment site of the superficial masseter muscle; the border line connects the notches ventrocaudal and craniodorsal to the mandibular angle. D: Alveolar cortical bone; the selection is defined by two lines drawn perpendicular to the lower border of the mandible, mesial and distal of the second molar.

For a detailed study of the bone characteristics, we used a micro-CT (μCT 40, Scanco Medical AG, Brüttisellen, Switzerland). During scanning all mandibles were similarly oriented and submerged in water to avoid dehydration. Scanning was performed at 10 μm spatial resolution and 45 kV peak voltage (effective energy: 24 keV). An integration time of 1200 ms was applied to substantially reduce noise and optimally discriminate between bone and background. An aluminum filter in the micro-CT and a correction algorithm reduced the effects of beam hardening [21]. A threshold of 642.8 mg hydroxyapatite/cm3 was used to distinguish bone from background. From the X-ray attenuation map, which contains the computed linear attenuation coefficients of each volume element (voxel) of the scan, mineralization of each voxel of the 3D reconstruction of the mandible was determined. From the 3D reconstructions the outer two voxel layers were peeled off to avoid the partial volume effects. For each mandible a distribution map of the cortical mineralization was obtained by projecting the mineral densities of all voxels in a 0.288-mm thick layer below the removed voxels on the surface of the mandible. A more detailed description of the method can be found in de Jong et al. [22]. Visual comparison showed possible local differences in surface mineralization degrees. Upon visual examination, various VOIs were determined selecting only the cortical bone at the attachment site of the masseter, the condylar head and below the second molar (Fig 1). For these VOIs, the cortical bone parameters (including bone volume, BV/TV and mineralization degree) were calculated taking into account the abovementioned threshold value. As mineralization was evaluated by including only "bone" voxels, the material density was calculated. Selection at the attachment site of the masseter and condyle is defined by the line connecting the two specific notches. For evaluation of alveolar cortical bone, the part of the second molar is picked up because of getting correct data from all mandibles.

#### Morphology

**Fig 2:**
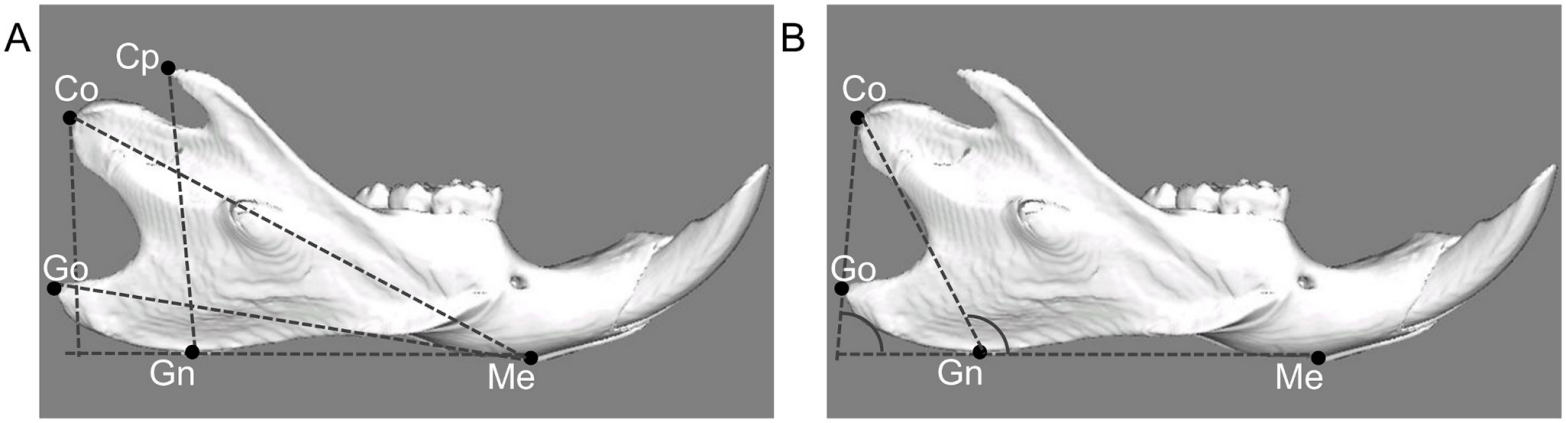
Landmarks and measurement items for linear analysis (A) and angular analysis (B) by 3D reconstructions. Me: Menton (most inferior point of mandibular symphysis). Gn: Gnathion (most anterior point on bony contour of mandibular symphysis). Go: Gonion (most outward and everted point on angle formed by junction of ramus and body of mandible). Co: Condylion (most superoposterior point on mandibular condyle). Cp: Coronoid process (most superior point on coronoid process of mandible). Me-Co: Total length of the mandible (distance measured between menton and condylion). Me-Go: Base length of the mandible (distance measured between menton and gonion). CpH: Height of coronoid process (a perpendicular line from coronoid process to the line connected to gnathion and menton). CoH: Height of mandibular ramus (a perpendicular line from condylion to the line connected to gnathion and menton). CoGo/GnMe: Gonial angle (angle made from the line connected to condylion and gonion and the line connected to gnathion and menton). CoGn/GnMe: Ramus angle (angle made from condylion, gnathion and menton).

For evaluating morphology, a CT- scan of similarly oriented mandibles was used (Latheta LCT-200, Hitachi Aloka Medical, Tokyo, Japan; 50kVp, 500μA, 48μm resolution). Three dimensional reconstructions of the mandibles were made for measurement of linear and angular dimensions (LEXI, Tokyo, Japan). Fig 2 shows the specified landmarks and measurements used in this study. Linear and angular dimensions were evaluated using five landmarks: menton (Me: most inferior point of mandibular symphysis), gnathion (Gn: most anterior point on bony contour of mandibular symphysis), gonion (Go: most outward and everted point on angle formed by junction of ramus and body of mandible), condylion (Co: most posterior and superior point on mandibular condyle), coronoid point (Cp: most superior point on coronoid process of mandible). In between these landmarks four linear and two angular measurements were assessed: total length of the mandible (Me-Co), base length of the mandible (Me-Go), height of coronoid process (Cp-GnMe), mandibular ramus height (Co-GnMe), gonial angle (CoGo/GnMe), and ramus angle (CoGn/GnMe).

#### Statistics

Average and standard deviation values were obtained for each experimental group. All data were tested for normality of distribution (Kolmogorov-Smirnov test) and uniformity (Bartlett’s test). Statistical analyses in this study were tested for differences using unpaired Student’s *t*-test. Probabilities of less than 0.05 were considered to be significant.

## Results

Animals in both groups showed an on average comparable increase of their body weight. Animals in the soft-diet group did not show any noticeable change in their daily use of their masticatory apparatus in response to the reduced food hardness.

### Mineralization

**Fig 3:**
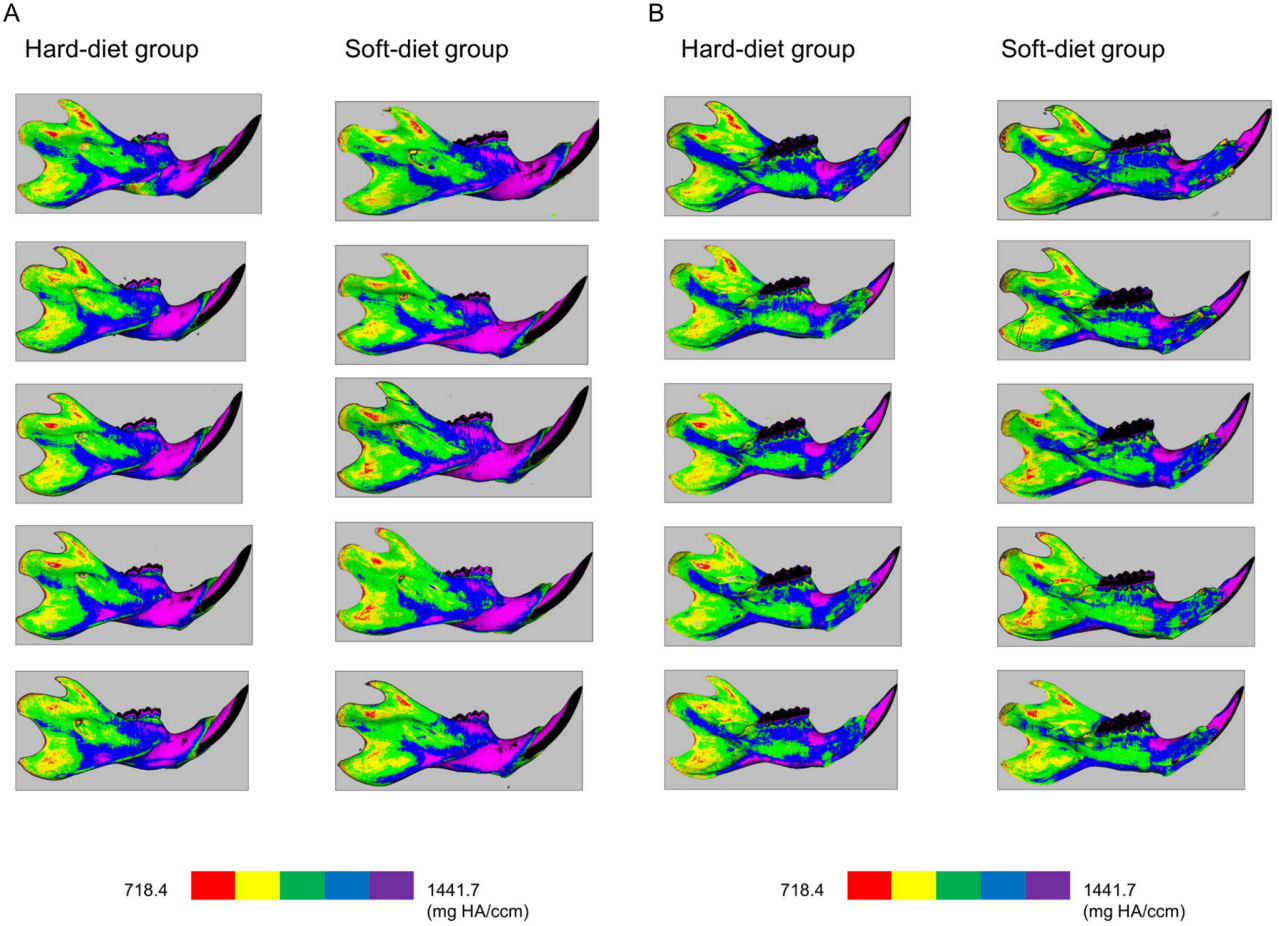
Lateral (3-A) and medial (3-B) views of the cortical mineralization. Left column: bones of the hard-diet group, Right column: bones of the soft-diet group. The colours red, yellow, green, blue, and purple indicate mineralization ranges 718.4–863.0, 863.0–1007.7, 1007.7–1152.4, 1152.4–1297.0, and 1297.0–1441.7 mg HA/ccm, respectively.

**Fig 4:**
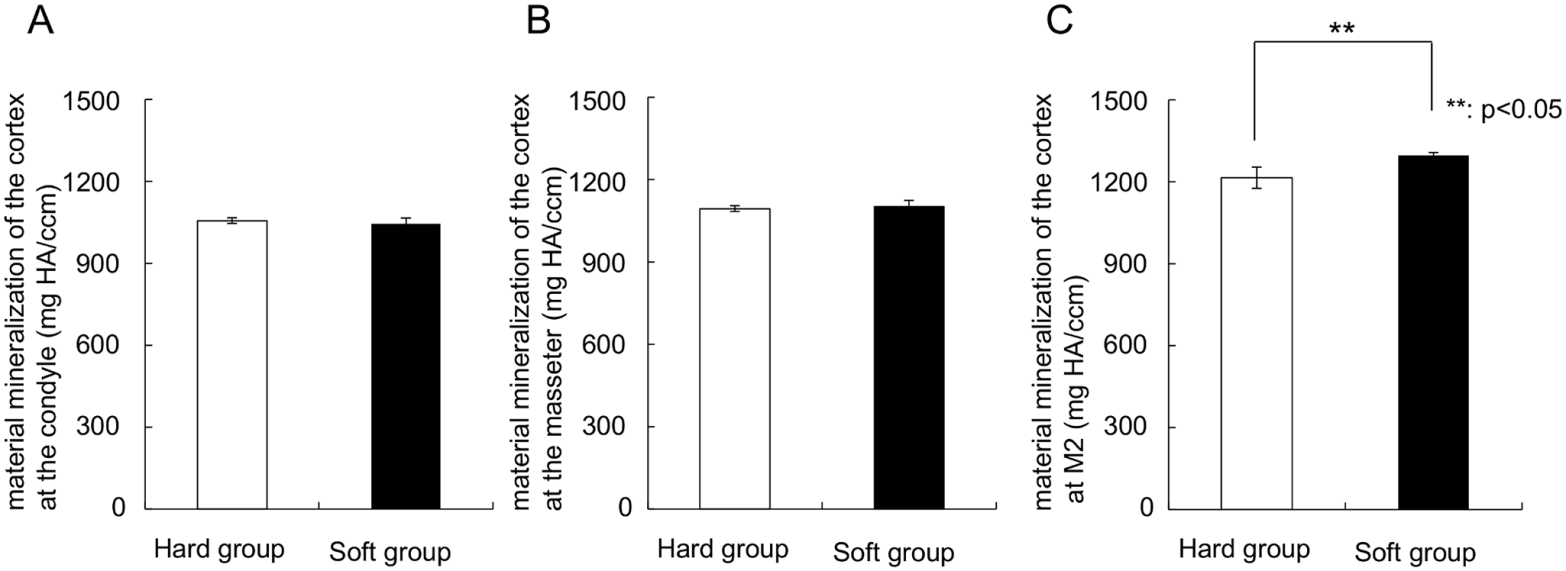
Graphs depicting the material mineralization of the cortex at the (A) Condyle, (B) Attachment of the masseter, (C) The part below the second tooth. **Significant difference between both groups (p<0.05).

Mineralization maps of the mandibles (Fig 3A and 3B) showed a remarkably similar distribution pattern of mineralization. Also, both groups showed very similar values and patterns in the regions of the condyle and the muscle attachments (Fig 4). However, below the dentition a significantly higher (p = 0.003) regional mineralization (material density) was found in the soft-diet group (1300 mg HA) compared to the hard-diet group (1200 mg HA) (Fig 4C).

### Bone volume and BV/TV

**Fig 5:**
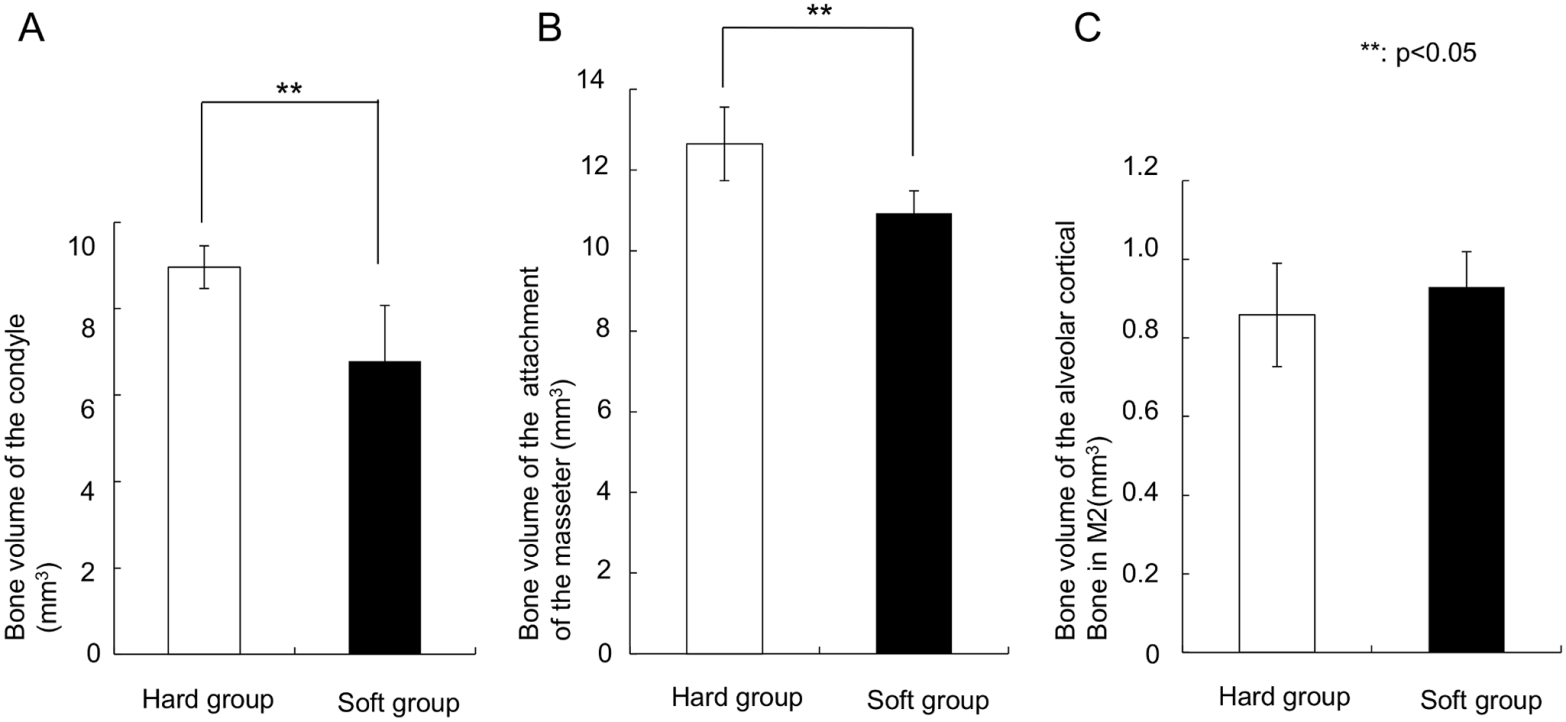
Graphs depicting the bone volume. (A) Condyle, (B) Attachment of the masseter, (C) The part below the second tooth. **Significant difference between both groups (p<0.05).

**Fig 6:**
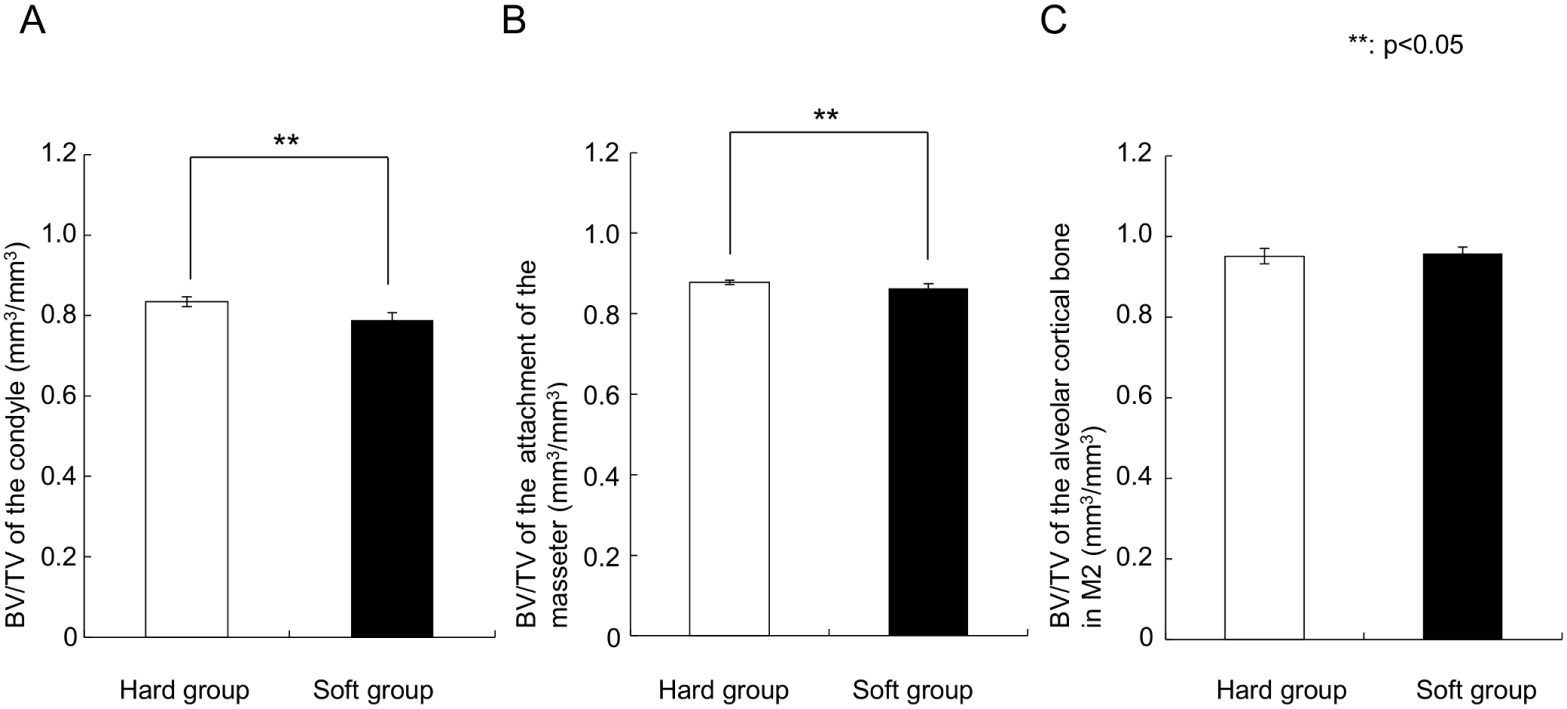
Graphs depicting the BV/TV. (A) Condyle, (B) Attachment of the masseter, (C) The part below the second tooth. **Significant difference between both groups (p<0.05).

The soft diet group showed a significantly (p = 0.01) lower bone volume in the condyle (6.8 ± 1.3 mm^3^) than the hard diet group (9.0 ± 0.5 mm^3^). Moreover, bone volume of the attachment of the masseter in the soft-diet group (10.9 ± 0.6 mm^3^) was also significantly lower than in the hard-diet group (12.6 ± 0.9 mm^3^) (Fig 5, p = 0.01). In contrast, the region of alveolar cortical bone did not show any difference in bone volume. In the same way, BV/TV of the condyle (0.789 ± 0.018 mm^3^ /mm^3^, p = 0.004) and the attachment of the masseter (0.863 ± 0.011 mm^3^/mm^3^, p = 0.042) in the soft-diet group showed significantly lower values than those in the hard diet group (0.834 ± 0.012 mm^3^/mm^3^ and 0.878 ± 0.005 mm^3^/mm^3^, respectively) (Fig 6).

### Morphometric findings

**Fig 7:**
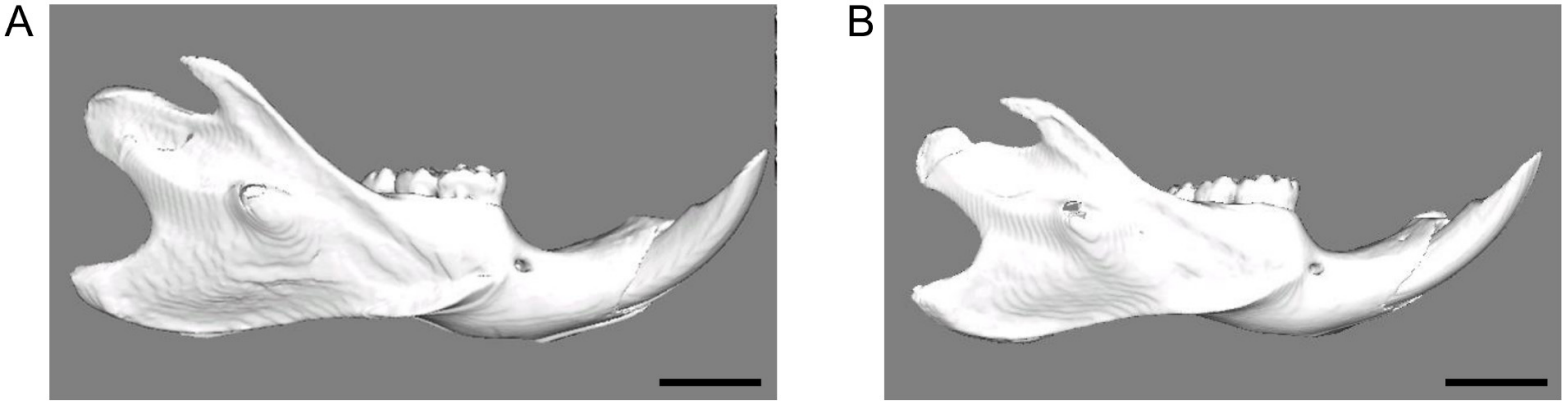
A representative 3D reconstructions of the hard-diet group (A) and the soft-diet group (B). Scale bar = 5 mm. Note the much smaller size of the soft-diet mandible.

Table 1 shows means and standard deviations for linear and angular measurements in both groups. The soft-diet group (9.70 ± 0.62 mm) demonstrated significantly (p = 0.007) shorter mandibular ramus height than the hard-diet group (11.14 ± 0.49 mm). Me-Co for soft-diet group also showed shorter length than for hard-diet group. Moreover, Fig 7 presents the 3D reconstructions for each group, and shows the overall smaller mandible of the soft-diet group compared to the hard-diet group. The mandibular gonial and ramus angles in the soft-diet group (mean ± s.d.) were 88.7 ± 2.8°and 112.2 ± 2.20°, respectively. These values were significantly larger (p = 0.008, 0.038), compared to the hard-diet group (82.2 ± 2.5°and 108.6 ± 1.8°).

**Table 1:** Comparison of mandibular measurements between the hard- and soft-diet groups.

Mandibular measurements		Hard group		Soft group		p-value
		Mean	S.D.	Mean	S.D.	
Linear measurements (mm)	Me-Co	22.06	0.34	21.14	0.70	0.05
	Me-Go	20.34	0.52	19.54	0.62	0.08
	Cp-GnMe	13.50	0.46	12.04	0.17	0.001>
	Co-GnMe	11.14	0.49	9.70	0.62	0.01
Angular measurements (°)	CoGo/GnMe	82.20	2.46	88.70	2.75	0.01
	CoGn/GnMe	108.60	1.81	112.20	2.20	0.04

## Discussion

The aim of this study was to evaluate the changes in mandibular bone due to decreased muscular loading caused by a soft diet during growth. The experimental results indicate that these changes are different for various mandibular regions. Compared to the hard-diet group, the mineralization degree was increased only at the occlusion region, while the bone volume and BV/TV was lowered at the condylar region and the attachment areas of the masseter. Apparently, a limited decrease in daily loading results in significant changes in bone characteristics. However, the changes in bone characteristics varied with location, including bone mass loss and increase in mineralization degree.

We already showed by using a telemetric system that a decrease in food consistency resulted in weaker jaw closer activity [23]. Simultaneously, the expression of insulin-like growth factor-1 (IGF-1), which is often used for evaluating the effect of mechanical load, decreased and the condylar growth was inhibited. In the present research, we can assume that the soft diet again resulted in lower muscle activity. The deficiency of masticatory demand during growth led to minor mandibular development. This inhibited growth resulted in less bone volume in the soft-diet group, although it didn’t show the correlation with mineralization.

In this study, the cancellous bone was not included because the masseter site did not contain any cancellous bone. At the other sites, the volume of the cancellous bone was also too little to get reliable means. As only the cortical bone was analyzed, bone mineralization was calculated as material density including only voxels exceeding the threshold value. As far as Fig 3 showed, the pattern of mineralization was very consistent and a clearly lower mineralization degree was found at the ramus compared to the corpus in both groups. This is quite similar to the pattern of mineralization found in rabbits by de Jong et al. [22]. They also found a very consistent mineralization pattern among used rabbits, and a lower mineralization degree at the ramus. As in mice, a major part of the ramus serves as an attachment site of the powerful jaw muscles. We can expect that this direct loading activates remodeling and creates lower mineralization compared to the corpus of the mandible, where much smaller and weaker muscles attach. Here, the occlusal force is the principle loading.

At the ramus, as mentioned above, many muscle forces are working, generated during various behaviors: grooming, licking, posture, yawning, chewing. As chewing is just one of the many loading patterns during daily life, a change in the muscle activity is probably not determining for the bone, as all other behaviors remain unaffected, generating equal amounts of loading. For the region below the molars, the only loading is when the teeth make contact (chewing). So, the loading pattern at the corpus is seriously affected by the soft diet. This may explain why, in the soft diet animals, the value of mineralization is unaffected at the ramus region, but increased at the region below the molars. Moreover, another possible factor for these results is the clearly defined VOI. Some previous studies showed remarkable regional differences in mineral density using a very small VOI [20, 24]. However, even more important than the size of the VOI is its location. As can be seen in Fig 3, the mineralization can vary enormously, even over very short distances. Choosing a VOI at a slightly different location in different animals can create large differences in the degree of mineralization. Therefore, the VOIs were standardized by clear reference points. A clearly defined location of measurement, as performed in this study, will minimize such errors.

The increased mineral content below the dentition seems to contrast the increased mineral content found in the seriously used tennis arm. However, there is a difference in time path. The increased mineral content in the occlusal area is probably a temporary state, caused by an acute decrease in remodeling rate due to the locally decreased loading regime. Eventually, the area will remodel towards a state that less bone volume will be present. The increased mineral content in the tennis arm is the result of years of training, and can be seen as an adaptation to the continuously increased use of the arm muscles.

The increased mineralization below the molars is in line with previous studies. The periodontal ligament is the connective tissue localized between the root of the tooth and the alveolar bone, and is known to react to mechanical loading such as occlusal force [25–27]. Several papers show the relation with loading and alveolar bone. In rats with reduced masticatory function by a soft diet, the alveolar bone of the mandible, was thinner [28, 29] and taller [28] and its bone density [28–30] was lower. Balazs et al. also indicated differences in the alveolar process and evidence that a reduction of occlusal loading induces a simultaneous response in both tissues [31]. This is in line with the increased mineralization below the teeth found in the current study.

For these experiments, ten rats were used. Although the sample size for each group looks small, we checked the minimum number of animals by the software named “G*Power 3.1.9.2” [32]. It followed that 5 rats per group is sufficient.

The results of the present study suggest that muscular loading is an important factor for bone characteristics and morphology during growth. Normal bone characteristics cannot be maintained after a limited decrease in daily loading. Also, the changes in bone characteristics are very region-specific, conceivably depending on the locally specific changes in the loading regime.

## References

[1] Nurmi-Lawton JA, Baxter-Jones AD, Mirwald RL, Bishop JA, Taylor P, Cooper C, et al (2004) Evidence of sustained skeletal benefits from impact-loading exercise in young females: a 3-year longitudinal study. J Bone Miner Res 19:314–322. 14969402 10.1359/JBMR.0301222

[2] Daly RM, Bass SL (2006) Lifetime sport and leisure activity participation is associated with greater bone size, quality and strength in older men. Osteoporos Int 17:1258–1267. 16680498 10.1007/s00198-006-0114-1

[3] Lang T, LeBlanc A, Evans H, Lu Y, Genant H, Yu A (2004) Cortical and trabecular bone mineral loss from the spine and hip in long duration spaceflight. J Bone Miner Res 19:1006–1012. 15125798 10.1359/JBMR.040307

[4] Watanabe Y, Ohshima H, Mizuno K, Sekiguchi C, Fukunaga M, Kohri K, et al (2004) Intravenous pamidronate prevents femoral bone loss and renal stone formation during 90-day bed rest. J Bone Miner Res 19:1771–1778. 15476576 10.1359/JBMR.040811

[5] Rubin CT, Lanyon LE (1985) Regulation of bone mass by mechanical strain magnitude. Calcif Tissue Int 37:411–417. 3930039 10.1007/BF02553711

[6] Cullen D M, Smith R T, Akhter M P (2001) Bone-loading response varies with strain magnitude and cycle number. J Appl Physiol 91:1971–1976. 11641332 10.1152/jappl.2001.91.5.1971

[7] Qin Y-X, Lam H, Ferreri S, Rubin C. Dynamic Skeletal Muscle Stimulation and its Potential in Bone Adaptation (2010) J Musculoskelet Neuronal Interact 10:12–24. 20190376 PMC4961074

[8] Burr DB (1997) Muscle strength, bone mass, and age-related bone loss. J Bone Miner Res 12:1547–1551. 9333114 10.1359/jbmr.1997.12.10.1547

[9] Turner CH (2000) Muscle-bone interactions, revisited. Bone 27:339–340. 10962342 10.1016/s8756-3282(00)00349-5

[10] Frost HM (2001) From Wolff's law to the Utah paradigm: insights about bone physiology and its clinical applications. Anat Rec 262:398–419. 11275971 10.1002/ar.1049

[11] Kannus P, Haapasalo H, Sievänen H, Oja P, Vuori I (1994) The site-specific effects of long-term unilateral activity on bone mineral density and content. Bone 15:279–284. 8068448 10.1016/8756-3282(94)90289-5

[12] Daly RM, Saxon L, Turner CH, Robling AG, Bass SL (2004) The relationship between muscle size and bone geometry during growth and in response to exercise. Bone 34:281–287. 14962806 10.1016/j.bone.2003.11.009

[13] Warner SE, Sanford DA, Becker BA, Bain SD, Srinivasan S, Gross TS (2006) Botox induced muscle paralysis rapidly degrades bone. Bone 38:257–264. 16185943 10.1016/j.bone.2005.08.009 PMC1435733

[14] Poliachik SL, Bain SD, Threet D, Huber P, Gross TS (2010) Transient muscle paralysis disrupts bone homeostasis by rapid degradation of bone morphology. Bone 46:18–23. 10.1016/j.bone.2009.10.025 19857614 PMC2818332

[15] Kiliaridis S, Mejersjo C, Thilander B (1989) Muscle function and craniofacial morphology: a clinical study in patients with myotonic dystrophy. Eur J Orthod 11:131–138. 2767145 10.1093/oxfordjournals.ejo.a035975

[16] Odman C, Kiliaridis S (1996) Masticatory muscle activity in myotonic dystrophy patients. J Oral Rehabil 23:5–10. 8850154 10.1111/j.1365-2842.1996.tb00804.x

[17] Zanoteli E, Yamashita HK, Suzuki H, Oliveira AS, Gabbai AA (2002) Temporomandibular joint and masticatory muscle involvement in myotonic dystrophy: a study by magnetic resonance imaging. Oral Surg Oral Med Oral Pathol Oral Radiol Endodont 94:262–271. 12221397 10.1067/moe.2002.124580

[18] Kawai N, Sano R, Korfage JAM, Nakamura S, Kinouchi N, Kawakami E, et al (2010) Adaptation of rat jaw muscle fibers in postnatal development with a different food consistency: an immunohistochemical and electromyographic study. J Anat 216:717–23. 10.1111/j.1469-7580.2010.01235.x 20579175 PMC2952384

[19] Yonemitsu I, Muramoto T, Soma K (2007) The influence of masseter activity on rat mandibular growth. Arch Oral Biol 52:487–493. 17126288 10.1016/j.archoralbio.2006.10.019

[20] Tanaka E, Sano R, Kawai N, Langenbach GE, Brugman P, Tanne K, et al (2007) Effect of food consistency on the degree of mineralization in the rat mandible. Ann Biomed Eng 35:1617–1621. 17522978 10.1007/s10439-007-9330-x

[21] Mulder L, Koolstra JH, van Eijden TMGJ (2004) Accuracy of microCT in the quantitative determination of the degree and distribution of mineralization in developing bone. Acta Radiol 45:769–777. 15624521 10.1080/02841850410008171

[22] de Jong WC, van Ruijven LJ, Brugman P, Langenbach GE (2013) Variation of the mineral density in cortical bone may serve to keep strain amplitudes within a physiological range. Bone 55:391–399. 10.1016/j.bone.2013.04.026 23659830

[23] Hichijo N, Kawai N, Mori H, Sano R, Ohnuki Y, Okumura S, et al (2014) Effects of the masticatory demand on the rat mandibular development. J Oral Rehabil 41:581–587. 10.1111/joor.12171 24702545

[24] Mavropoulos A, Kiliaridis S, Bresin A, Ammann P. (2004) Effect of different masticatory functional and mechanical demands on the structural adaptation of the mandibular alveolar bone in young growing rats. Bone 35:191–197. 15207756 10.1016/j.bone.2004.03.020

[25] Kusters ST, Kuijpers-Jagtman AM, Maltha JC (1991) An experimental study in dogs of transseptal fiber arrangement between teeth which have emerged in rotated or non-rotated positions. J Dent Res 70:192–197. 1999558 10.1177/00220345910700030701

[26] Shuttleworth CA, Smalley JW (1983) Periodontal ligament. Int Rev Connect Tissue Res 10:211–247. 6358097 10.1016/b978-0-12-363710-9.50010-1

[27] Enlow DH (1990) Physiologic tooth movements and alveolar remodeling In: Enlow DH, ed. Facial growth. Philadelphia: Saunders, pp130–148.

[28] Mavropoulos A, Odman A, Ammann P, Kiliaridis S (2010) Rehabilitation of masticatory function improves the alveolar bone architecture of the mandible in adult rats. Bone 47:687–692. 10.1016/j.bone.2010.06.025 20601301

[29] Bresin A, Kiliaridis S, Strid KG (1999) Effect of masticatory function on the internal bone structure in the mandible of the growing rat. Eur J Oral Sci 107:35–44. 10102749 10.1046/j.0909-8836.1999.eos107107.x

[30] Sato H, Kawamura A, Yamaguchi M, Kasai K (2005) Relationship between masticatory function and internal structure of the mandible based on computed tomography findings. Am J Orthod Dentofacial Orthop 128:766–773. 16360919 10.1016/j.ajodo.2005.05.046

[31] Denes BJ, Mavropoulos A, Bresin A, Kiliaridis S (2013) Influence of masticatory hypofunction on the alveolar bone and the molar periodontal ligament space in the rat maxilla. Eur J Oral Sci 121:532–537. 10.1111/eos.12092 24206071

[32] Faul F, Erdfelder E, Lang AG, Buchner A (2007) G*Power 3: a flexible statistical power analysis program for the social, behavioral, and biomedical sciences. Behav Res Methods 39:175–191. 17695343 10.3758/bf03193146

